# THE RELATIONSHIP BETWEEN STATE MEDICAID HCBS PROGRAMS AND FAMILY CAREGIVING

**DOI:** 10.1093/geroni/igae098.1047

**Published:** 2024-12-31

**Authors:** Johanna Thunell, Elise Parrish, Katherine Miller

**Affiliations:** University of Southern California, Los Angeles, California, United States; University of Pennsylvania, Philadelphia, Pennsylvania, United States; Johns Hopkins University, Baltimore, Maryland, United States

## Abstract

A growing number of persons are opting to age in place in home- and community-based settings. As demand for care in these settings increases, the supply of caregivers – both family and paid care – is not keeping pace. Yet, programs providing financial payments, education, and training for family caregivers may increase the likelihood of successfully aging in place. As no national long-term care insurance system exists in the US, most home- and community-based services (HCBS) are paid out-of-pocket or through Medicaid HCBS waivers. Through waivers, some Medicaid beneficiaries can pay family caregivers for care received. However, payment availability and eligibility criteria vary within and across states. We leverage the geographic-restricted NHATS data (2011–2019) to identify a cohort of respondents with at least one functional limitation. Using multinomial logistic regression, we estimated the relationship between living in state in a year with a Medicaid waiver providing payments to family caregivers and the likelihood of receiving: paid and family care, paid care only, family care only, or no care. After adjusting for demographics and family structure, we found respondents living in a state allowing for payments to family caregivers were 2.2 percentage points more likely to receive care by a family member only and 1.3 percentage points less likely to receive formal care only. Unpacking the mechanisms by which policies can incentivize (or reduce) caregiving has implications for broader policy discussions to (1) ensure an adequate workforce to meet the demand for care and (2) mitigate adverse consequences of family caregiving.

